# Juggling the Limits of Lucidity: Searching for Cognitive Constraints in Lucid Dream Motor Practice: 4 Case Reports

**DOI:** 10.3390/brainsci15080879

**Published:** 2025-08-18

**Authors:** Emma Peters, Clarita Bonamino, Kathrin Fischer, Daniel Erlacher

**Affiliations:** 1 Institute of Sport Science, University of Bern, Bremgartenstrasse 145, 3012 Bern, Switzerland; emma.peters@unibe.ch (E.P.);; 2 School of Exercise and Nutrition Sciences, Queensland University of Technology, Victoria Park Road, Kelvin Grove, QLD 4059, Australia; clarita.bonamino@gmail.com

**Keywords:** lucid dreaming, dream control, motor practice, dreaming, REM sleep

## Abstract

Background/Objectives: Lucid dreaming (LD), during which the dreamer becomes aware of the dream state, offers a unique opportunity for a variety of applications, including motor practice, personal well-being, and nightmare therapy. However, these applications largely depend on the dreamer’s ability to control their dreams. While LD research has traditionally focused on induction techniques to increase dream frequency, the equally important skill of dream control remains largely underexplored. This study provides an exploration into the mechanisms of LD motor practice, dream control, and its potential influencing factors. We specifically examined whether a complex motor skill—juggling—could be performed during LD, calling for relatively high levels of dream control and access to procedural memory. Methods: Four healthy participants underwent overnight polysomnography (PSG), provided detailed dream reports, and completed questionnaires assessing dream control and self-efficacy. Dream-task success was assessed using predefined in-dream motor performance criteria. Differences between high and low LD control participants were examined, and two detailed case reports of lucid dream juggling attempts provide insight into the challenges of executing complex motor tasks during LD. Results: Dream control varied between and within participants. Both dream control and self-efficacy seemed to predict participants’ ability to execute the LD motor task. Conclusions: Despite the low sample size, this study highlights the potential roles of individual traits like self-efficacy in shaping dream control abilities and motor performance during LD. By using empirical, task-based measures, this study helps build the foundation for future research aimed at optimizing LD applications in clinical and non-clinical fields.

## 1. Introduction

Motor imagery, the mental rehearsal of actions without physical execution, activates neural circuits involved in movement control and has been shown to improve performance across domains such as sports and rehabilitation [1,2]. Combining physical and mental training often enhances outcomes more than either alone [3]. This suggests that mentally simulated movements may support motor learning when physical practice is not possible.

Lucid dreaming (LD), where one is aware of being in a dream, offers a unique extension of mental practice by allowing movements to be rehearsed within dreams in an immersive, virtual-reality-like environment [4,5]. Movements performed during LD activate brain regions involved in motor execution, as shown through functional Magnetic Resonance Imaging (fMRI) [6]. Early studies confirmed that dream-enacted movements produce subtle physiological signals, despite Rapid Eye Movement (REM) sleep atonia [7,8]. Further research shows that movement planning in lucid dreams resembles waking motor control. For example, sensorimotor area (SMA) activation during dreamed hand movements was found using electrophysiology (EEG) [9] and fMRI [6]; this SMA activation is comparable to physical execution. The SMA plays a key role in movement preparation, suggesting that LD practice could enhance real-world motor skills by fine-tuning the movement preparation.

Motor rehearsal during LD has shown promise in enhancing waking performance [4,5]. Prior research has explored how naturally occurring distractions within the dream may have a detrimental effect on lucid dream motor practice [10]. In a dart-throwing task, researchers observed that both the level of dream control and the presence of distractions affected performance outcomes. One participant, for example, reported interference from a dream character: “The doll kept throwing darts at me” (p. 2367). Another noted instability in the dream environment: “I noticed it was getting somewhat unstable … I managed three or four more throws and then I woke up” (p. 2367). These findings support the use of LD as a space for embodied cognitive rehearsal but also highlight that LD is often unstable. Dreamers may struggle with maintaining stability, controlling dream environments, or sustaining lucidity [11]. These challenges may reflect the interplay between bottom-up processes, such as automatic sensorimotor activations for successful movement execution, and top-down regulation for dream control involving cognitive control, metacognition, and belief systems. While some dream actions may be supported by procedural memory, broader dream control likely depends on higher-order executive functions. Despite LD’s potential for motor practice, a major challenge lies in the lack of control over the dream environment and the unpredictability of dream scenarios. The ability to manipulate dream elements and perform complex tasks varies widely among individuals due to differences in cognitive abilities, dream recall, and dream stability [11,12]. Some lucid dreamers report being able to solve mathematical problems, play instruments, or juggle, whereas others struggle to exert basic control over their dreams [11,13]. This distinction between lucid insight (awareness of dreaming) and control (modifying the dream itself) suggests that awareness alone does not guarantee the ability to alter the dream world. Dream control challenges are further evident in lucid nightmares, where dreamers remain aware but experience reduced agency and emotional distress [14]. Common features include feelings of helplessness, aggressive dream characters, and difficulties in waking up.

Although several dream control techniques have been proposed [12,15], the lack of empirical evidence makes it unclear whether dream control is a trainable skill that can be systematically developed or if it is inherently constrained by individual differences in cognitive and personality traits. One potential factor influencing dream control is self-efficacy, which refers to an individual’s belief in their ability to achieve specific goals [16]. While qualitative data suggest that self-efficacy may influence dream control [17], questionnaire findings indicate that self-efficacy does not predict LD skills (such as awareness and control) [18]. These mixed findings suggest that the role of self-efficacy in dream control abilities remains unclear. While some research suggests that self-confidence positively correlates with frequent LD [19], no direct evidence has yet confirmed a causal relationship between self-efficacy and dream control. However, having more lucid dreams may not necessarily correspond to greater dream control. While frequent lucid dreamers recall dreams more vividly, there is no conclusive evidence that they have greater control over dream content than rarer lucid dreamers. Yet, many lucid dreamers report difficulty carrying out intended tasks due to insufficient clarity or fading lucidity [11]. Lucidity itself often fluctuates within the same lucid dream, existing on a continuum rather than a fixed state, ranging from minimal dream awareness to full lucidity [20]. Dream control also appears to vary along a comparable spectrum, with higher levels of lucidity generally associated with greater dream control [21].

Overall, the factors that facilitate higher control and sustain dream lucidity remain poorly understood. A questionnaire study by Sammer et al. [18] found that only LD frequency and prior knowledge of LD predicted LD skills (a composite measure of awareness and control). Other factors, including age, dream recall, nightmare frequency, sleep quality, meta-awareness, self-efficacy, induction technique use, dream journal use, and beliefs related to LD, were not significant predictors. More research is needed to determine which variables optimize dream control. Identifying the specific cognitive and emotional traits, such as meta-awareness, self-efficacy, attentional control, and stress resilience, that underpin dream control is important to help unlock the potential of LD in areas such as mental health, trauma therapy, motor skill training, and creative problem-solving.

This study systematically investigates lucid dream control through the performance of a complex motor task—juggling—within a lucid dream. Juggling was selected due to its relatively high difficulty in the LD context, requiring not only procedural motor coordination, but also specific volitional dream control including dream stabilization, object manipulation, and regulation of dream physics (e.g., gravity). The study also investigates how individual differences in self-efficacy may influence the capacity for both dream control and motor execution during LD. By using a relatively demanding motor task in a lucid dream, the study aims to explore how individuals regulate dream environments while attempting a pre-determined coordinated action thereby advancing the understanding of the mechanisms involved in dream control during LD motor execution.

## 2. Materials and Methods

### 2.1. Study Design

This study invited lucid dreamers to spend a minimum of one night in the sleep laboratory at the University of Bern to practice a motor task within a lucid dream. Data collection included dream reports and measures of waking and dream-related juggling skills and self-efficacy. Participants who successfully lucid dreamed on the first night but were unable to complete the experiment (e.g., practice the motor task) were invited for additional sessions to reattempt it.

### 2.2. Participant Recruitment and Selection

The sample consisted of four participants (*n* female = 1, mean age: 24, SD: 1.0) recruited from the Institute for Sports Science (University of Bern), via social media advertisement, personal connections, and posters on campus. Exclusion criteria included any sleep-related disorders and an LD frequency of less than 2–3 a year. Signed consent was obtained prior to data collection and ethical approval was obtained from the Ethics Commission of the Faculty of Humanities (2022-01-00008) of the University of Bern. Table 1 displays the dream recall and LD frequency, juggling skills, and confidence in juggling for each participant.

### 2.3. Instruments

#### 2.3.1. Online Questionnaire Measures

Prior to their first sleep laboratory session, participants completed an online questionnaire via LimeSurvey (in English). The survey included demographic questions, as well as several psychometric scales. Dream recall frequency was assessed using Schredl’s [22] 7-point scale ranging from 0 (never) to 6 (almost every morning). LD frequency was rated on a 5-point Likert-type scale ranging from 1 (less than once/month) to 5 (more than 5 times/week). Participants also rated their waking juggling skills using a 5-point Likert-type scale (1 = minimal proficiency, 5 = exceptional proficiency). Self-efficacy for juggling was measured separately for the waking and dream states using Bandura’s [23] 11-point confidence scale (0 = cannot do at all, 100 = highly certain can do). Specifically, participants were asked to rate how certain they were in their ability to execute juggling in the lucid dream and in wakefulness. These self-efficacy ratings reflect participants’ beliefs in their ability, not whether they had previously attempted the task in a lucid dream. Dream control was assessed using a custom-developed 5-point Likert-type scale asking participants to rate their perceived control over various dream elements including control over their own dream body and actions, other characters, dream objects, dream environment, time, and gravity (1 = low control; 5 = high control). As this scale is exploratory in nature and not yet validated, this limitation is noted in the discussion. The online questionnaire items can be found in Supplementary Material S3.

#### 2.3.2. Polysomnography

Participants were monitored using a 10-channel polysomnography (PSG) setup, including EEG (F3, F4, C3, C4, O1, O2), horizontal electrooculography (EOG), electromyographic (EMG) (chin), a reference electrode (Fz), and a ground electrode (A2, right earlobe). Electrodes were connected to a gUSBamp amplifier, following the standardized 10–20 EEG system [24]. PSG was conducted throughout the night for a separate analysis (not reported in the present study). For the purpose of this study, PSG data were solely used to confirm that participants were physiologically asleep and to identify sleep stages.

### 2.4. Procedure and Protocol

Participants were instructed to abstain from caffeine or alcohol for the 24 h prior to each sleep laboratory session. At 21:00, they prepared for bed and PSG electrodes were applied. Participants then went to bed and were encouraged to fall asleep. Participants slept in fully dark, windowless rooms with no light exposure during the night. No light stimulation was used, and lighting conditions were consistent throughout all sessions. Upon identification of N2 sleep, a timer was set for approximately four hours, during which participants were allowed to sleep undisturbed. They were then awakened and immediately provided a verbal dream report (see Supplementary Material S1 for the dream report questions), which was audio recorded at the bedside to minimize dream memory loss. If lucidity was not achieved during this time, a “wake back to bed” (WBTB) protocol was implemented. During the 30-minute WBTB, participants reported prior dreams and discussed their LD goals and preferred induction methods. Each participant was instructed to attempt juggling within the lucid dream using three balls and to remember this goal upon achieving lucidity. This task was reinforced as the central LD objective for the night. After this 30-minute period, participants returned to sleep and attempted to induce lucidity using their preferred technique. They were subsequently awakened after a REM period and a dream report was collected. Only one participant (P2) reported a lucid dream prior to the WBTB; all others became lucid afterward. If participants had practiced juggling during the lucid dream, they subsequently rated their task-specific self-efficacy using Bandura’s [23] single-item 0–100 confidence scale (0 = cannot do at all, 100 = highly certain can do). This was completed immediately after the verbal dream report. Three participants completed this rating following lucid dreams involving juggling attempts. As part of the dream report, participants were prompted to provide detailed descriptions of how lucidity was attained and the experience of practicing the motor task (e.g., preparing the environment, procuring juggling balls, level of dream control, difficulties encountered).

### 2.5. Data Analysis

Questionnaire data was exported from LimeSurvey and handled in Excel. Due to the small sample size, no statistical analyses were conducted; the data are solely reported descriptively. Dream reports were collected in German, Swiss German, and English. To ensure consistency and clarity, all reports were standardized following the dream reporting manual by Schredl [13]. Reports originally collected in German or Swiss German were then translated into English using the DeepL translation tool [25] and manually reviewed to correct potential inaccuracies and ensure fidelity to the original content.

Participants were assigned to either the high lucid dream control (LDC) group (*n* = 2) or the low/no lucid dream control (NLDC) group (*n* = 2) based on scores from the custom-developed 7-item dream control scale (see Section 2.3.1). Grouping was determined by participants’ control ratings over their own dream body and dream body movement, which were the only items that could be consistently evaluated across all participants. Those in the LDC group reported higher levels of control (range: 3–5), while the NLDC group showed low control (range: 0–2). Other scale items (e.g., control over dream characters or objects) were excluded from the grouping decision when they could not be rated due to their absence in the dream content. In addition to group comparisons, detailed dream reports of the two participants in the LDC group are presented to provide deeper insights into their LD experiences and control mechanisms.

## 3. Results

### 3.1. Online Questionnaire

Results from the online questionnaire regarding participants’ self-reported levels of self-efficacy, dream control, challenges with dream control, and experience in performing tasks of different natures in their lucid dreams are depicted in Section 3.1.1, Section 3.1.2, Section 3.1.3 and Section 3.1.4 respectively. This data offers insights into the nature and the extent of dream control experiences prior to the in-lab part of the study. The full scores for the online questionnaire can be found in the following Supplementary Tables: Supplementary Tables S4.1, S4.2, S4.3, S4.4, S4.5, S4.6, S4.7, and S4.8.

#### 3.1.1. Self-Efficacy

Participants’ self-efficacy in the LD and waking state is presented in Table 2. Overall, the LDC group (P1 and P2) reported higher self-efficacy in juggling in both states compared to the NLDC group (P3 and P4).

#### 3.1.2. Dream Control

Questionnaire data indicated that all participants reported having previously attempted to control their lucid dreams:

- NLDC group: Changing dream scenery or environment (*n* = 2), interacting with dream characters (*n* = 1), manipulating objects or events (*n* = 0), flying or levitation (*n* = 1), and passive observation (*n* = 2).
- LDC group: Changing dream scenery or environment (*n* = 1), interacting with dream characters one, manipulating objects or events (*n* = 1), flying or levitation (*n* = 2), and passive observation (*n* = 2).

Table 3 shows average and individual ratings of dream control abilities, comparing the LDC and NLDC groups. Both groups rated control over their own dream body and movements highest, and control over other dream characters and time the lowest.

#### 3.1.3. Challenges in Controlling Aspects of Lucid Dreams

Both participants in the LDC group reported encountering difficulties across nearly all aspects of their lucid dreams, including maintaining lucidity, remembering goals, allowing supernatural events to unfold, staying focused, imagining supernatural actions, and distinguishing dreams from reality. Participants in the NLDC group experienced challenges in maintaining lucidity, but none reported difficulties in remembering goals or imagining supernatural actions.

Participants in the LDC and NLDC groups differed in the challenges they faced when manipulating dream elements:

LDCgroup: Participants reported increased difficulty with greater dream control influence, especially when attempting new, unfamiliar, or supernatural actions. Successfully changing the dream environment required vivid mental imagery or prior familiarity with the location. Control over dream characters was limited. Both participants found altering time perception nearly impossible.

NLDCgroup: Participants reported limited control, typically restricted to their own body movements and minor object manipulations. Engaging in supernatural actions disrupted gravity, making it unpredictable or unstable. Altering the dream environment was considered difficult and only possible when pre-existing mental images or concepts were available.

#### 3.1.4. Successful Task Completion

Table 4 displays the number of participants in each group who reported successfully completing specific tasks during a lucid dream. In the LDC group, both participants reported achieving four out of the eight actions, indicating higher overall control. In contrast, the NLDC group reported fewer successful task completions, with most actions completed by either one or no participant. Additionally, P2 (LDC group) reported being able to transform into an animal.

### 3.2. In-Laboratory Dreams—Full Sample Results

Overall, all participants had at least one lucid dream in the lab, with P1 experiencing a total of three. Table 5 compares different aspects of dream juggling attempts for the LDC and NLDC groups.

### 3.3. In-Lab Dream Control Ratings–Full Sample Results

Table 6 presents individual ratings of dream control for each participant. P1 reported high control ratings (ratings of 4 or 5) for all aspects except for dream character control (rating of 1). P2 reported high ratings of dream control only for the dream body and movements (rating of 5), but ratings of 0 for the remaining aspects. The NLDC group generally reported low levels of control, with ratings ranging from 0 to 3.

A clear distinction emerged between the two groups: the two participants in the LDC group consistently reported higher levels of control, with ratings predominantly of 4 and 5, while the two participants in the NLDC group mostly rated their control as low, with scores of 1 and 2.

### 3.4. In-Laboratory Dreams—Juggling Case Studies

This section provides a descriptive analysis of the lucid dreams experienced by the LDC group. P1 and P2 reported high dream control and attempted juggling during LD. The full dream reports can be found in the Supplementary Material S2.

#### 3.4.1. Participant 1 (P1)

P1 had extensive LD experience, with an LD frequency of several times per week and a dream recall of almost every night. He typically induced lucidity using WBTB and often became lucid by recognizing anomalies within his dreams. P1 completed three separate sleep lab sessions with all lucid dreams occurring after the WBTB protocol. On night one, he reported a dream within a dream with active decision-making and conversations but no juggling attempt. He rated his moderate control for shaping the environment and influencing gravity (3/5), but low for body movements and interactions with characters (1/5). On night two, P1 reported several short lucid dreams, often becoming lucid upon noticing unusually large crowds—a common cue for him. He then proceeded to attempt juggling. “As I was aware that I was lucid, I consciously set goals, for example juggling”. (P1). He initially could not find juggling balls, so he began searching for them. Once found, he felt nervous or sensed resistance from the dream environment, so he only held the balls:

“I wanted to juggle, but I couldn’t because I didn’t have balls... Once I had balls, I don’t know if I was nervous, or the dream world didn’t want me to juggle. Then I actually just had the balls in my hand”.

He then experienced a false awakening, dreaming of repeatedly falling off the bed until the researcher appeared to help him. This was followed by another false awakening where the researcher reappeared with oranges, prompting him to juggle:

“I dove back into a dream and that’s when you were there again. Because I was hungry, you had oranges and then I showed you how I could juggle”.

In his lucid dream, he reported showing advanced juggling skills. Initially, he tossed one ball between his hands, progressed to juggling three in a circular motion, and finally controlled them telepathically, causing them to move in a snake-like pattern:

“I threw the ball normally back and forth... then I tried... three balls in a circle... then I threw the balls back and forth with my mind, snake-like”.

He reported being able to change the balls’ weight based on throwing speed. However, when he became aware that this defied real-world physics, control collapsed, and the balls fell. Sometimes, he experienced a conflict between his lucid intention and dream resistance:

“Sometimes I was aware that I was dreaming, but my body didn’t want to... I have the goal now, but until I reached the goal, it took a little bit”.

He also described tunnel vision in the dream, which helped him stay focused and maintain control.

P1 rated his juggling performance with traditional balls as 2/5 and with oranges as 4.5/5. Dream control for manipulating objects and gravity was rated at 4/5, dream body and environment control over his body at 3/5, and dream character interaction at 2/5.

On night three, P1 experienced a short lucid dream where he created a scene in which he was drinking coffee with a friend but lost dream control shortly thereafter:

“I created a scene. Afterwards I had no control, there was not more than one scene either”.

He reported experiencing some difficulties with dream stability eventually waking up.

“The more I wanted to change or create, it wasn’t stable afterwards, I woke up right after”.

Despite the significantly short lucid dream, he reported a very high control rating—control over the dream body, movements, environment, and gravity at 5/5, and control over objects at 4/5. He reported choosing not to exert control over dream characters, rating this aspect at 1/5.

#### 3.4.2. Participant 2 (P2)

P2 completed two separate nights at the sleep laboratory. He was unable to attain lucidity on the first night. On night two, he experienced one lucid dream, in which he practiced juggling by mimicking juggling movements with his hands, but without any juggling balls. However, his practice was interrupted by a dream character:

“… I did the (juggling) movement right away … You (the researcher) turned to me and put your hands in front of my eyes. You were trying to stop me from doing something”.

P2 recognized that his lucid dream was going to be short, which motivated him to attempt juggling quickly. Even though he had no balls, he initiated the juggling movement, hoping that balls might appear—but they did not:

“I thought that maybe the balls would come, but they didn’t. I thought, ‘Since I can do it myself, it won’t be much different.’ So, I just did the movement”.

P2 described experiencing full clarity during the dream and feeling joy upon realizing he was lucid. The overall LD experience was perceived as realistic and comparable to waking life, including his juggling skills, body movements, and the effects of gravity. He reported full control over his body, but was unable to rate his level of control over dream characters, objects, or gravity as he did not attempt to influence them.

## 4. Discussion

This study explored how lucid dreamers might regulate their dreams when instructed to execute a juggling motor task. Additionally, it considered the potential influence of self-efficacy on dream control during motor practice in LD. The study revealed variability in participants’ ability to control dream content, particularly when performing a goal-directed, complex motor task such as juggling. Success varied both between and within individuals, and was influenced by factors such as self-efficacy, familiarity with the task, and perceived plausibility of dream actions. Participants with higher waking and dream confidence in their juggling ability tended to perform the LD task better. Unrealistic or unfamiliar manipulations often destabilized the dream or proved more difficult to execute. Execution of a juggling task may be considered, within the context of LD, relatively challenging as it not only requires precise motor execution but also specific volitional dream control including dream stabilization, object manipulation, and management of physics within the dream (e.g., gravity). This dual demand provided an opportunity to observe how bottom-up activation of procedural, sensorimotor memory (the juggling movement) may interact with top-down volitional dream regulation in a high-complexity setting. Consistent with previous research [11,21], participants showed significant between- and within-person variability in their ability to control various aspects of their lucid dreams, including the environment, actions, their own bodies, and dream physics. Further, successful motor practice of the juggling tasks depended on their ability to control these elements. While LD has been proposed as a tool for motor training [4,5], success appears to depend heavily on the dreamer’s ability to exert control and deal with distractions within the dream [10]. These findings support the view that achieving lucidity alone does not guarantee control over specific dream components.

As expected, juggling posed a challenge to participants, particularly due to unpredictable dream physics. Participants often struggled to initiate or maintain control over the juggling motion. These difficulties may be tied to factors like self-efficacy, meta-awareness, and emotional projection [15,17]. Participants with a stronger belief in their juggling ability—both in waking life and in dreams—tended to better execute the dream task. Three out of four participants rated their LD dream juggling ability as higher than in waking life, with both participants in the LDC group reporting maximum confidence (100%). We interpret this performance not as the result of procedural memory alone, but as the outcome of a hybrid process: bottom-up reactivation of sensorimotor skills interacting with top-down dream control mechanisms, including the ability to maintain dream stability, regulate expectation, and modulate the environment. Successful motor execution may rely on bottom-up processes involving automatic sensorimotor activation and procedural memory—similar to the replay of well-learned physical skills. In contrast, dream control refers to the volitional manipulation of dream content (e.g., objects, characters, spatial or narrative structure) and likely requires top-down regulation involving higher-order executive functions such as cognitive flexibility, inhibitory control, and metacognitive insight. These processes likely recruit frontoparietal brain networks associated with conscious self-regulation and metacognition which may not necessarily be strengthened through task repetition alone. Thus, dream control and motor task execution may rely on distinct cognitive systems. Achieving both concurrently—especially in service of specific performance goals—may demand extensive training and practice, as observed in contemplative traditions such as Tibetan dream yoga [26]. Training a motor skill during LD, therefore, does not only involve practicing the task itself, but also learning to operate within a dynamic, non-physical, and often unstable environment.

According to Bandura’s [23] self-efficacy theory, belief in one’s ability enhances performance; thus higher self-efficacy could in turn support better LD motor performance and potentially, overall dream control. However, evidence is mixed. A questionnaire study of 344 lucid dreamers found no correlation between self-efficacy and LD skills, including control [18]. Conversely, qualitative accounts suggest that self-doubt undermines dream control attempts whereas confidence supports success [17]. These conflicting findings may be due to methodological differences or perhaps also different interpretations of the concept of self-efficacy. For instance, someone may believe in their capacity to control dreams but still report low dream control based on recent failures. Conversely, they might rate their confidence as low but recall moderate success. Such discrepancies highlight a potential dissociation between perceived and actual dream control. It is important to distinguish between self-efficacy related to a specific task and broader dream control abilities. Participants with high waking self-efficacy in juggling may perform better in dream-based juggling attempts due to procedural memory and task familiarity, rather than enhanced volitional control over the dream environment. As such, task-specific self-efficacy may serve more as a motivational or preparatory factor than a direct indicator of general dream control. This distinction reflects the difference between bottom-up (e.g., motor skill reactivation) and top-down (e.g., volitional manipulation of dream content) mechanisms. The present study attempted to address this by assigning a concrete task (juggling) during in-lab LD episodes, suggesting a potential link between self-efficacy and dream-task performance in goal-directed dream scenarios. Our findings highlight the importance of distinguishing between task performance rooted in procedural memory and dream control that involves top-down volitional modulation. The ability to perform a known motor task in a dream, such as juggling, may largely rely on bottom-up reactivation of established sensorimotor pathways during REM sleep. In contrast, dream control, as defined by the ability to manipulate the dream environment, narrative, and laws of physics, likely involves cognitive flexibility, metacognitive insight, and frontal executive function. These aspects were not directly measured in the current study, and we emphasize that successful motor performance in LD should not be conflated with high-level dream control.

Dream control varied widely: while some participants were able to modify environments and perform complex actions, others struggled to maintain lucidity or act intentionally. Interestingly, LDC participants—despite their greater success—also faced more challenges, possibly due to the increased cognitive load associated with altering additional or complex aspects of the dream world while sustaining lucidity. A consistent difficulty across participants was generating novel dream elements. Familiar modifications (e.g., changing known settings or interacting with familiar characters) were easier than creating new or abstract content. Larger-scale changes often destabilized the dream, suggesting limits imposed by cognitive or emotional constraints. To manage these challenges, participants often adopted a “go with the flow” approach, avoiding direct interactions that might cause instability. Even in lucid states, participants’ belief systems constrained dream control. Tasks perceived as unrealistic (e.g., telekinetically moving juggling balls) proved more difficult. Indeed, participants showed better success with plausible actions (e.g., juggling balls with hands) than with those that defy waking-world physics, implying that cognitive schemas from waking life persist in dreams, shaping perceived possibilities. When an action is deemed implausible, dream control often diminishes [21]. Similar patterns have recently been observed in an adolescent sample, where pre-conceptions of reality appeared to limit dream control [17]. These findings align with Mallett and colleagues’ [21] view that dream bizarreness and lucidity coexist in a complex relationship: highly bizarre dreams may enhance lucidity but not necessarily facilitate control. For LD motor training to be effective, dreamers may need to retrain their assumptions about what is possible in a dream. Future studies could explore progressive exposure to increasingly implausible dream actions, potentially expanding dreamers’ control range by gradually overriding waking-world constraints.

Participants’ experiences support the notion that both lucidity and control exist on a continuum, in line with current perspectives [21]. NLDC participants generally adopted a more passive stance, while LDC participants pursued more ambitious dream manipulations, sometimes at the cost of dream stability. Successful control attempts included initiating and maintaining conversations, altering lighting conditions (turning day into night), flying, and environmental transformations. Despite their unrealistic nature, these activities retained elements of physical experience, possibly making them easier to conceptualize and enact.

Due to the small sample size, inferential statistics were not feasible, limiting the generalizability of the findings. Recruiting suitable participants proved to be particularly challenging, and only half practiced juggling in the laboratory. Low recruitment rates and difficulties in achieving lucidity in laboratory conditions are common occurrences in the field [27]. One further limitation is that prior experience or attempts at juggling, either during lucid dreams or in wakefulness, were not assessed. While self-efficacy ratings reflect participants’ beliefs, prior LD or waking experience with the task could have influenced their perceived or actual dream performance. Lastly, participants in the NLDC group experienced, on average, less than one lucid dream per month. Future research could benefit from more stringent inclusion criteria, such as requiring a minimum LD frequency of one lucid dream per week, to enhance the likelihood of collecting sufficient in-lab LD reports.

## 5. Conclusions

Unlike studies relying on subjective ratings or interviews, this study objectively measured task-specific dream control by assigning participants a defined motor task (juggling) during LD episodes. This method allowed for a direct examination of motor execution in lucid dreams. Findings suggest that dream control may develop over time. Participants with more LD experience and greater control also faced more challenges, likely because they attempted more demanding actions. This raises a key question: Can dream control be systematically trained? If repeated LD exposure helps manage increasingly complex tasks, targeted training could enhance dream control. Future studies should test whether structured LD practice and cognitive strategies (e.g., belief modification, visualization, expectation-setting, dream yoga) can improve dream performance. For example, lucid dream yoga, rooted in contemplative sleep practices, is designed to train awareness and may be used to improve control within dreams [28]. Clarifying how psychological and cognitive factors shape control can help define predictors of dream success and inform the design of LD interventions across domains.

Dream control during LD plays an important role in allowing individuals to pursue goals and intentions within their dreams. This study provides initial insights into how self-efficacy may relate to the execution of complex motor tasks in lucid dreams. By assigning a concrete task—juggling—during in-lab LD episodes, findings highlighted the challenges and variability inherent in dream control, shaped not only by lucidity but also by beliefs and prior experience. While dream control holds promise as a tool for motor practice, our findings show that lucidity alone is not sufficient; instead, dreamers’ perceived task plausibility and waking-world schemas significantly constrain or enable control.

## Figures and Tables

**Table 1:** Sample Characteristics.

Participant No	Gender	Dream Recall Frequency	Lucid Dreaming Frequency	Waking Juggling Skills Self-Rating
1	M	5	3–5 times per week	2
2	M	5	1–3 times per month	5
3	M	5	Less than once per month	1
4	F	6	Less than once per month	2

Note. Dream Recall Frequency: 5 = several times a week; 6 = almost every morning Schredl [22]. Waking Juggling Skills Self-Rating measured on a 5-point Likert-type scale (1 = minimal proficiency, 5 = exceptional proficiency).

**Table 2:** Self-efficacy in juggling in the lucid dreaming and waking state.

Self-Efficacy		
	On a scale from 0 to 100, how certain are you that you can successfully execute juggling in	
Participant	Lucid Dream	Wakefulness
1	100	50
2	100	100
3	40	20
4	60	20

Note. Ratings during lucid dreaming and wakefulness. Scores range from 0 (cannot do at all) to 100 (highly certain can do) [23].

**Table 3:** Dream control rating by group (LDC vs. NLDC).

‘Do You Have the Ability to Control in Your Lucid Dreams?’	LDC (*n* = 2)	NLDC (*n* = 2)
…own dream body	4 (3, 5)	1.5 (2, 1)
…own movements	4 (3, 5)	1 (0, 2)
…equipment	2 (4, 0)	1.5 (2, 1)
…environment	1.5 (3, 0)	1 (1, 1)
…other dream characters	1 (2, 0)	1.5 (1, 2)
…gravity	2 (4, 0)	2 (3, 1)

Note. Values are group averages with individual scores in parentheses: LDC = average (P1, P2), NLDC = average (P3, P4). Ratings range from 1 (low control) to 5 (high control).

**Table 4:** Successful task completion by group.

What are the Challenges You Have Already Managed to Do in a Lucid Dream?	LDC Group (*n* = 2)	NLDC Group (*n* = 2)
Communicate with dream characters	2	2
Deliberately shape dream environment	1	1
Flying with full control	2	2
Make day turn to night	2	1
Going through walls or objects	1	0
Going through dream characters	1	1
Eat food	2	1
Transform into an animal *	1	0

* Category identified from the questionnaire’s open-ended option.

**Table 5:** In-laboratory dream juggling attempts by group.

Reported Feature	LDC	NLDC
Attaining Lucidity	By noticing anomalies. P1. Noticed unusually large crowds. P2. Performed a reality check by inspecting his right hand: “Then I looked again more closely, and the ring finger or index finger was missing. I just had four fingers”.	By noticing anomalies, being informed by dream characters. P3. “It’s always like as if someone would knock and say: ‘hey, you’re actually dreaming!’” P4. “I realised I was dreaming because I felt asleep strangely”.
Juggling Attempts	Managed to practice the task. P1. Initially struggled executing the task with juggling balls but later succeeded with three oranges. Performance ratings: 2/5 with balls, 4/5 with oranges. P2. Mimed the movements without juggling balls/objects. Unable to rate dreamed skill performance in dream report.	No juggling attempts. P3. Waited for external guidance and never thought of juggling. P4. Considered juggling but did not attempt it.
Dream Stability	Varied dream stability P1. Their dream initially became unstable but it re-stabilized when a relevant dream character (the researcher) appeared. P2. Initially stable, their dream became unstable when the researcher (dream character) covered their eyes, triggering awakening.	Varied dream stability P3. Fluctuations in both stability and awareness. P4. High dream stability but low awareness.
Intentions and Goals	Set clear goals before going to sleep. P1 and P2. Remembered their intention of juggling and were able to achieve it to some extent (see juggling attempts).	Set clear goals before going to sleep P3. Forgot the goal. P4. Remembered the intention of juggling but was interrupted before attempting it.
Laws of Physics	Varied. P1. Loss of gravity: “The whole room shook, and things suddenly flew around. It became quite intense”. Further irregularities with the law of physics in a following lucid dream: “I realized that the physics were not right at all”. P2. No complications; physics experienced as realistic.	Varied. P3. Unintentionally broke the laws of physics by floating and rotating in the air while meditating. P4. No complications; physics experienced as realistic.

**Table 6:** Individual ratings of in-lab dream control by participant.

‘How Much Control Did You Have Over…’	LDC Group		NLDC Group	
	P1	P2	P3	P4
…your own body in the dream	3	5	1	1
…your movements	3	5	0	2
…the equipment/object in your dream	4	0	2	1
…the environment in your dream	3.7	0	1	1
…other dream characters	2	0	1	2
…gravity/physics	4	0	3	1

Note. Rated on a 6-point scale (0 = no control, 5 = high level of control). P1′s ratings are averaged across his three lucid dreams.

## Data Availability

The original contributions presented in this study are included in the article/Supplementary Materials. Further inquiries can be directed to the corresponding author.

## Supplementary Materials

The following supporting information can be downloaded at: https://www.mdpi.com/article/10.3390/brainsci15080879/s1, Supplementary S1: Dream Report Questionnaire; Supplementary S2; Translated and Converted Dream Reports; Supplementary S3: Online Questionnaire. Table S4.1: Demographics, Table S4.2: Dream control 1, Table S4.3: Dream control 2, Table S4.4: Dream Control 3, Table S4.5: Dream Control 4, Table S4.6: Dream Control 5, Table S4.7: Dream control 6, Table S4.8: Dream Control 7.
