# Supplementary material for: Juggling the Limits of Lucidity: Searching for Cognitive Constraints in Lucid Dream Motor Practice: 4 Case Reports
Source: Brain Sci. 2025 Aug 18;15(8):879. doi: 10.3390/brainsci15080879 (PMC12384728; doi:10.3390/brainsci15080879)
Supplement: Supplementary file 1 [file brainsci-15-00879-s001.zip › brainsci-3776086-supplementary.pdf]

Supplementary Material

Supplementary S1: Lucid Dream Skill Questionnaire (LUSK) Results

Table S1. Lucid Dream Skill Questionnaire (LUSK) Results

				In how many of your lucid dreams...									
Participant	Age	Gender	LD Frequency	Aware of differences to the waking state (e.g., bizarre incidents or settings)?	Aware that your physical body was asleep	Aware that all dream objects were not real	Thought about different options of what you can do in a dream	Able to keep your awareness for a satisfying period of time	Decided deliberately to observe the dream as a dream	Shape your environment (e.g., change landscapes/surroundings, let	Have full control of your dream body (movements, actions)	Chose deliberately a specific action?	Perform specific actions (e.g., flying, floating, talking with dream characters, perform
1	25	male	Several times a week	In half	In none	In none	In all	In a quarter	In half	In half	In half	In half	In half
2	25	male	Two or three times a month	In three quarters	In three quarters	In three quarters	In three quarters	In three quarters	In a quarter	In half	In half	In three quarters	In three quarters
3	24	male	About once a week	In three quarters	In half	In a quarter	In none	In half	In all	In a quarter	In three quarters	In all	In all
4	24	female	About two to four times a year	In half	In half	In a quarter	In half	In a quarter	In a quarter	In a quarter	In a quarter	In half	In half

Note. LD = Lucid Dreaming

Supplementary S2: Dream Report Questionnaire

Open Question: What went through your head before you woke up?

Specific Questions:

On a scale from 1-5, how clear was your dream?

- Did you become lucid?
- How did you become aware that you were dreaming?
- Could you give the eye signal?
- Did you have control over the dream content?
- Did you try to juggle?
- On a scale of 1-5, how good were your juggling skills?
- With what kind of object or item did you juggle?
- Did you have a clear intention or goal for the dream?
- How vivid was the dream environment?
- Were you able to in interact with dream characters and objects as if they were real?
- Did you experience any negative emotions during the dream?
- How long did the lucid dream last?
- Did you dream in color?
- Were the physics in the dream normal?
- Did time move normal?
- Was the dream stable?

Motor task related questions:

Rate these items from,1-5: How much control did you have over...

- ... own body in the dream?
- ... your own movements?
- ... the equipment/objects in your dream?
- ... the environment of your dream?
- ... other dream characters?
- ... The physics/gravity of the dream?

S3: Translated and Converted Dream Reports

Participant 1 - Dream report 1

You woke me up but just before at the very end I realized that I am still in the dream. In this scene I was already once. It was in the sleep lab, but the sleep lab was not here but in Italy, Spain area on a hill. Next to the hill was a house protected by the military. We did experiments, but you still have a father and mother who are with the mafia. He doesn't want you to do experiments, and that's why the lab is hidden at a like miniature golf, similar setup with roller coaster hidden in the eighteenth hole, with a giant mill. It was my task to lucid dream and that's why I was by my bed and fell asleep I almost made it once and the second time I made it in the dream. Then I didn't even wake up and you came in, woke me up, but it was in the dream of the dream. You woke me up and I woke up. He told me that it was good, you can take a break now, go out for a run, but you have to be careful and because I didn't have any pants, and you gave me a pair. I had to be inconspicuous. We went out with your father and mother. I wanted to go back to the lab, but I didn't have a key. You had to be inconspicuous like one, beggar would give the money, you handed it to me and your father noticed the pants because they were so perfectly tailored for him, and he noticed because they are his pants. I imitated certain poses like a beggar and then he said yes go. To go back it was bright because it was like in a battlefield. On a hill like a castle or hut, I had to overcome this first. There had formed some group of villagers. They were not good, sometimes they shot themselves or they could not handle the gun. I kept running on the side towards the sleep lab. Then I could sleep again. Before I got to the sleep lab. I was on the bus to get here; I saw a couple of assistants who were also on the bus, and they knew a better way to get to the sleep lab. He said you must get off earlier and then I got off before and it was a new scenery, Italian vibes. I realized I was in a dream and told the kid to wait a minute. I moved aside to make the sign quickly. It was a very unfamiliar sign to make and continue dreaming, quickly I no longer knew how to do it. Then I tried a few times and noticed that I was getting a little more awake and went back to dreaming. Then I was already in the sleep lab, it went on with the dream within the dream. Before you woke me up, I was in some kind of sleep paralysis? I was in that state, you actually had to wake me up a little bit more effectively. The bus scene was only short, I just gave the signal and let it continue. I got to the sleep lab thinking I was already awake. Once I started dreaming again, I had a little

more control. Before the dream of the dream, I dreamed that I was in the lab and not specially equipped because the whole room shook, and things suddenly flew around it became quite intense. You were supposed to wake me up for real, I had no control. In the second dream I had more control, in the communication between you and me I consciously moved away from you a little bit because of your father. To come back to the lab, I had to choose a path and just give direction but effectively what I do, I sometimes backed off and left what was coming.

Participant 1 - Dream report 2

The first dreams were about juggling, I was in the dream, and I realized that I was in the dream. Then I first tried to give the sign and then I wanted to juggle, but I couldn't because I didn't have balls and then I had to go looking for balls once I had balls, I don't know if I was nervous, or the dream world didn't want me to juggle. Then I actually just had the balls in my hand. Certain things in the dream I knew were not true and then I thought: 'aha'. In the beginning I always thought all dreams were real until I realized the difference, that can't be true. For example, in the dream there were way too many people who are actually not there at that time. Then I thought I was waking up, but in the end, I was in a dream and I had already fallen on the floor a few times, but I didn't fall on the floor at all, I was always in bed. I juggled with normal juggling balls. In the beginning I only had, even though I was looking for three, I only tried with one or two because I can't juggle properly. Once I managed, but it was not on the first or second try, but on the third, with oranges. I didn't have to look for them, I found them because I was hungry. The process of waking up, I actually didn't wake up from the dream, I just kept dreaming. I thought I had fallen off the bed, that wasn't true at all, because then you came and picked me up off the floor again, but then I thought that can't be true, and woke up and as I fell asleep again, I dove back into a dream and that's when you were there again. Because I was hungry, you had oranges and then I showed you how I could juggle. As soon as I was aware that I was lucid, I had consciously set some goals, for example juggling. But there were always some difficulties that sometimes I was only lucid for a short time. As soon as I wanted to do something that the dream wouldn't allow, I had to improvise a little. When I was not lucid, it was very clear with certain details. Once I became lucid, the focus zoomed in and on small details and the big hole was no longer there or at least blurred. At the beginning I was nervous, exited, then a certain fear when, for example, I fell off the bed and still couldn't get up, like I was paralyzed and had to wait for you. But then when you came over. It went on forever. Already several days, two days. For example, in the beginning the gravity was crazy because sometimes when I juggle, I throw the ball normally back and forth in two hands and then I tried, because it was easy, three balls in a circle one after the other. Because that also worked, I threw the balls back and forth with my mind snake-like. That also worked and then I realized that the physics is not right at all, and the balls usually fell to the ground. Weight of the ball I could determine in each case according to

the speed when throwing, that it becomes lighter. It did not make sense afterwards and as soon as I thought it did not make sense, I could control it afterwards in the dream. Other things happened around me, because it did not work afterwards. Sometimes it took me a little while to realize it was a dream. It always felt very real until I realized that couldn't be true. Sometimes I was aware that I was dreaming, but my body didn't want to. It was more influenced by the dream world, but I was aware, I have the goal now but until I reached the goal it took a little bit. The things I was controlling, balls and such, I was already very focused on that. There was also the dream world focused into it. I didn't have to look at the whole world anymore and then there's more control. I looked for a setting and then it happened automatically and I didn't have to change much afterwards. When I was looking for the balls or trying to juggle I had to set a lot of things in motion and that gave a lot back. The more I changed things, the harder it became to juggle.

Participant 1 - Dream report 3

It was a housewarming party of a colleague; we supported her there. I crept back and forth, watched and supported the one colleague to change the lamp. The one who calls her did not show up, then I had to support. The colleague's name is *colleague*. The people introduced themselves, then I said that I am *Participant 1*. I saw another triplet. Then I could only consciously approach next to that particular scene, it was very fast until I woke up again. Only in that one particular scene. I created a scene, afterwards I had no control, there was not more than one scene either. It was there, in a room, a table, the scene with the coffee drinking and the one colleague who was standing there. The lucid one wasn't very vivid, it was just the two of us. It was just empty. At the housewarming party it was very lively and very many people, familiar and unfamiliar faces. In the lucid dream, in the beginning there were only two of us and I deliberately created the coffee scene. I started talking to her, but this was very short, the dream, and it was after with a lot of interaction, there has been communication, but not verbal rather with actions and eye signs. Body language. With the other one, there has been a lot of talking. In the beginning I was in a main hall, suddenly *colleague* came out of the ceiling and said: "yes *Participant 1*, can you help me quickly assemble the lamp" and I so "yes". When I was done, I went outside and there were a lot of people gathered on the sofa. The house was very big, and *colleague*'s mother came and introduced herself to the people. Swords, her brother and they all looked exactly the same from the face. I don't know her

family at all, but I know the mother and they all had the mother's face, but tall, short, fat, it was funny. The sister introduced triplets she has, but no sister has all the triplets. Meanwhile, I woke up. In the lucid dream with the colleague, I go for a coffee today, I was a little nervous I would say. At the housewarming party I had some nostalgia of meeting people from the past and some joy. The first one had the people, me and her, they didn't look at me, but she looked like she used to, a little bit colored but the environment was white because I created that. The second one, it was super-duper multicolored. She was not coming down from the ceiling, she was in the ceiling and opening up the ceiling. Before she opened the ceiling, I was alone in that room. Because I heard a noise. Then I was walking back and forth to see where the noise was coming from and then suddenly, she opened the blanket and said: "*Participant 1*, can you quickly…" she first asked where the other colleague was, I asked her "probably outside smoking" and that she said, "help me quickly" and I was like "okay". There the gravity was all right. Overall time would have been normal. But certain scenes went faster in that sense, others were in a normal time frame. The perception of me was like in real life, sometimes things pass faster because you are fully in the conversation and sometimes, I thought: 'when this is finally over?' I had the feeling that the short dream when I was lucid felt much slower, that time was not passing like in the dream now. The more I wanted to change or create, it wasn't stable afterwards.

Participant 2 - Dream report 1

Did you receive my signal? I gave it several times. I had a lucid dream and did the juggling movement. You were sitting next to me doing something, I looked at my hand and I had four fingers. I did the eye movement quickly, left right left right. I did it several times. Then I did the movement right away. While I was doing it, I kept doing LRLR in between. You turned to me and put your hands in front of my eyes: "laugh". You were trying to stop me from doing something. Then it was over. I meant I woke up because you came into the room and I asked, "hey did you get it?" you said "yes". Then we high fived. In this case, this was still a dream too. I even ripped the electrodes off here, funny. I looked at my right hand and it didn't seem like a dream at all. Then I looked again more closely, and the ring finger or index finger was missing. I just had four fingers. Then I noticed it. It was only a short section, but there I had control. There I was lucid. I did the eye sign right away, remembered it. Then started juggling. I wanted to start it really fast, not that I find distractions. I think at first that in the course, balls would come but they did not. I thought, but because I can do it myself, it won't be much different. I thought I'll just do the movement. It was just the left side, which I perceived because I was sitting down. You were in front of me doing something at the table with the cables. I was looking down. I was sitting to the left, that's all I perceived. I was simply carrying out the intention, not much else. The moment of surprise that I look at the hand. Joy in the sense of "Yeh" and "okay, okay, I have to do this now". It felt like I didn't have much time, and I wanted to do as much as possible in that time. It was not the stability. Like it was going down, that moment when the person turned around and covered my eyes, I knew it was slowly over. I didn't even think about moving anywhere else. The sequence was not so long. Maybe half a minute. From the first time LRLR to the last LRLR, I was steadily making the juggling motion. It had normal color. It was bright, the wall was white. What you were wearing I don't know; I think it was a dark shirt. I can't tell clearly. There wasn't much gravitational stuff going on either. I just sat there and made arm movements. It didn't have any balls, unfortunately.

Participant 3 - Dream report 1

I was here, I had to get up. I didn't realize it was a dream; I thought I had to leave, then we talked. I left because I thought I was dreaming and then I went to the desert and dreamed of

meditating. Then I tried to do something with my eyes, but I don't know if I managed it. I didn't want to influence or try to influence the dream. I just know that afterwards we ate a pineapple that weighed 13kg. I kept trying to move my eyes. I was meditating on a balcony on a desert in a palace. I was turning with my body. I always thought I had to move my eyes. Whether it worked or not, I don't know. At the very beginning I dreamt that you woke me up and then you said that we have to go now, then I sat. Someone has to take the things off my head. You came back and took the things away then we talked. You told me that you fancy me, I said that I noticed this, and we should kiss once. But it won't have a future I said and now I have to pack my stuff and go. Then another guy I knew came in. He asked what I was still doing here, I should pack my stuff. Then I was standing here in the kitchen and I still had to wash my stuff. That was the moment when I realized "Yes, you are dreaming, you haven't left yet, and you are still lying here in bed". Then the switch took place when I was suddenly meditating in the desert. The beginning with getting up felt very real and towards the end it was unreal. The one in the desert was like a narration, as if you were listening to a famous story from a meditation story. Then it was surreal, it became more and more surreal. It's always like this, the thought flies through my head as if someone would knock on it and say "hey, you're actually dreaming". It's exactly the same as if someone said "hey, you're dreaming". I say it to myself, but I don't know what impulse makes it happen. When I talked to you, I had feelings of love and closeness, but also some disappointment towards you, it felt weird. I felt guilty towards you, and you said it was all okay, but I didn't really believe that. Then we were okay, and I left it on the side and was washing up. At the end of the story, I was emotionless. The dream happened, as if by itself and I tried to influence, I didn't specifically try to do anything. My only goal was to move my eyes, I didn't know why anymore. Juggling didn't cross my mind. I was just moving my eyes and then waiting for someone to tell me what to do. When I was meditating, it was already in the desert in the palace, the people, I don't know if it was me or not, I was like floating and I could turn while floating. Then when I had the thought that I had to move my eyes, I also turned in the meditation position.

Participant 4 - Dream report 1

I had the feeling that I was half aware that I was dreaming at one point, because it was so long and complicated. I also dreamed about the laboratory. There were a lot of people here.

First, I woke up as a test person, then there were all these people around, I half recognized them, they were all from a school. I wanted to go back to sleep, but the people were doing something, they were having a seminar in this room I was in. Another room opened up, there were still a few playing basketball. "laughed" I actually wanted to go back to sleep and then we all went for a walk together. It was all very realistic. It all made sense. Because everything was in the lab, I didn't feel like I was dreaming. Then I fell asleep again in my dream and then I realized that I was dreaming but it was too weird, I couldn't really do anything. I actually went to sleep in a dream and then, while falling asleep, I realized that I was dreaming because I fell asleep strangely and woke up again and then was awake really quickly. I had the intention in my dream but thought I was awake and dreamed about the same situation in my dream. I was disturbed by quite a lot of people in my dream. I was actually trying to juggle. I didn't get to it. I first dreamed of the laboratory, and everything was very vivid. Now it comes to my mind, there was a little dog. I was petting it and talking to people, it was all very lively. Then we went for a walk, it was all very realistic and very detailed. I talked with quite a lot of people and also with you, you were there too. I discussed with people, with one of them I even, now it comes back to me, I was in the lab, and I wanted to go to sleep and there were people sitting on my bed and I had to shoo them away and the one I had to push away a little bit because he was lying on my bed. My bed was in the middle of the room and there were lots of desks all around, like a seminar room. They wanted to do a session there and I wanted to go to sleep, they wanted to be there, I said "no I want to go to sleep!". Then we made a compromise that we would all go for a walk. When I wanted to go to sleep, I was angry at the people for all of them being in my room. Then I argued with them, I thought it was unfair, I tried to show them that it is very important to me that I can go back to sleep now, they should wait a few more hours. I was looking for sympathy, I was a little disappointed and angry at people. Afterwards I also had positive emotions when they were more empathetic after all, and we looked for a solution. When I woke up in my dream, I felt like it was time for WBTB and I was awake for the whole morning, it felt like it was 2-3 hours. It was very stable, I was not directly aware that it was a dream, so I was just dreaming, and I experienced everything strongly. It was very stable.

Supplementary S4: Online Questionnaire

Lucid Dreaming Questionnaire

1. Personal Information:

First name:

Lastname.:

Age:

Gender:

Occupation:

- o Student
- o Retired
- o Füll-time employee
- o Part-time employee
- o Self-employed
- o Unemployed
- o Others:

2. Juggling:

How good would you describe your juggling skills on a scale from 1-5? (1 not good at all, 5 very good)

Can you juggle with three objects? (yes/no)

How confident are you in your juggle skills on a scale from 1-5? (1 not confident at all, 5 extremely confident)

3. Dream Recall:

How often do you remember your dreams in the last few months?

- o Almost every morning
- o Several times a week

- About once a week
- 2 to 3 times a month
- About once a month
- Less than once a month
- Not at all

4. Lucid Dreaming Experience:

Have you ever experienced a lucid dream before? (yes/no) How frequently da you typically have lucid dreams?

- Rarely (less than once per month)
- Occasionally (1-3 times per month)
- Moderately (1-2 times per week)
- Frequently (3-5 times per week)
- Very frequently (more than 5 times per week)

5. Dream Control:

How do you usually behave when you become lucid? (Select all that apply)

- Changing the dream scenery or environment
- Interacting with dream characters
- Manipulating objects or events
- Flying or levitation
- Just observe.
- Other (please specify)

Have you ever attempted to control or manipulate the events in your dreams? (yes/no)

On a scale of 1-5, how would you rate your ability to control your dreams regarding the following items? (1 not .successful at all, 5 extremely successful)

- Own dream body
- Other dream characters' bodies

- Own dream body movement
- Own actions
- Other dream characters' actions
- Objects
- Environment
- Gravity

What challenges or limitations have you experienced in controlling aspects of your dreams? (Select all that apply)

- Maintain lucidity.
- Remembering your goals
- loosing focus
- lmagining yourself doing something supernatural
- Having difficulty distinguishing between dreams and real-life situations
- Others

In controlling your dream, what is the challenge with...

...your own dream bodies?

....other dream character's' bodies?

....your own dream body movement?

....your own dream actions?

... other dream characters' actions7

... the objects in your dream?

... the dream environment in your dream?

....time in your dream?

....gravity in your dream?

How much do the challenges you mentioned above affect the control over your dream? Rate the challenges of each item on a scale from 1-5. (1 - no effect on the dream control at all, 5 - affects the dream control extremely)

What are challenges you already managed to do in a lucid dream? (Select all that apply)

- o Communicate with other people in your dream.
- o Deliberately shape your environment
- o Flying with full control
- o Make day turn to night.
- o Going through walls, or things
- o Going through dream characters
- o Eat food.
- o Others

6. Motivation and Persistence:

7. Self-Efficacy

How certain are you that you can successfully execute juggling in a lucid dream?

Rate your degree of confidence by recording a number from O to 100 using the scale below.

(O = extremely uncertain; 100 = extremely certain)

How certain are you that you can successfully execute juggling in the waking state"?

Rate your degree of confidence by recording a number from O to 100 using the scale below.

(O = extremely uncertain; 100 = extremely certain)

Rate your degree of confidence by recording a number from O to 100 using the scale below.

Cannot do at all (0) Moderately can do (50) Highly certain can do (100)

Supplementary S5: Results of the Online Questionnaire

Table S5.1 Demographics

	Personal		Juggling			Dream recall	
Participant	age	gender	juggling skills from 1-5?	Can you juggle with three objects?	confident juggle skills from 1-5?	How often remember dreams in the last few months?	How do you usually become aware that you are dreaming?
1	25	male	2	Yes	2	Several times a week	Dream signs or anomalies
2	25	male	5	Yes	5	Several times a week	Intention setting before sleep
3	24	male	1	No	2	Several times a week	when something doesn't suit me in my dream
4	24	female	2	Yes	2	Almost every morning	Spontaneous realization

Table S5.2 Dream control 1

	How do you usually behave when you become lucid? (Select all that apply)					
Participant	Changing the dream scenery or environment	Interacting with dream characters	Manipulating objects or events	Flying or levitation	Just observe	Other
1	Yes	Yes	Yes	Yes	Yes	Create a world starting with just a white blanket room
2	No	No	No	Yes	Yes	
3	Yes	Yes	No	No	Yes	
4	Yes	No	No	Yes	Yes	

Table S5.3 Dream control 2

On a scale of 1-5, how would you rate your ability to control your dreams regarding the following items? 1 - not successful at all 2 - once in a while successful 3 - sometimes successful 4 - mostly successful 5 - extremely successful

	Have you ever attempted to control or manipulate the events in your dreams?								
Participant	Own dream body	Other dream characters' bodies	Own dream body movement	Own actions	Other dream characters' actions	Objects	Environment	Time	Gravity
1	4	3	4	4	3	4	4	2	4
2	4	2	3	4	1	3	3	3	2
3	4	1	5	5	2	3	3	1	2
4	2	1	3	2	1	1	2	1	2

Table S5.4 Dream control 3

	What challenges or limitations have you experienced in controlling aspects of your dreams? (Select all that apply)						
Participant	Maintaining lucidity	Remembering your goals	Letting supernatural things happen	Loosing focus	Imagining yourself doing something supernatural	Having difficulty distinguishing between dreams and real-life situations	Other
1	Yes	Yes	Yes	Yes	Yes	Yes	
2	Yes	No	Yes	No	Yes	No	
3	Yes	No	Yes	No	No	Yes	
4	Yes	No	No	Yes	No	No	

Table S5.5 Dream control 4

	What is the challenge in controlling... Write down the challenges you experience when trying to control the following item. Use a short explanation.				
Participant	…your own dream body?	…other dream characters' bodies?	… your own dream body movement?	… your own dream actions?	… other dream characters' actions?
1	Normalerweise kein Problem, aber desto komplexer der Traum, desto schwieriger habe die Kontrolle über mein Körper.	Gleich wie "own dream body", mit Ausnahme mein Gehirn lässt nicht gern kontrollieren (nach dem erschaffen), da ich meistens an den "outcome" interessiert bin.. also meistens sind es Charakter die ich im Alltag kenne und deswegen obwohl ich sie kontrollieren könnte. will ich es nicht (ethisch Blockade.. in mein Gehirn)	Normalerweise kein Problem, aber neue Sachen lerne, die man vorher nie im TV, bücher und oder selbstgemacht hat.. steigt die Schwierigkeitsgrad	Kleine Änderungen gehen gut, kompliziert Prozesse noch schwierigkeiten	Mache es nicht gern, mit Ausnahmen (falls Gefahr besteht..)
2	To be lucid enough	They look like own characters	To be lucid enough	To remember my intention	To believe that it is possible, asking them kindly
3	full control of my body	never do this	all movments, the same as in reality	take, throw, influence things	never controls other pers.
4	imagening it	I don't know how to control them	sometimes I can't move at all.	Sometimes I can only watch what is happening and can't	I can only influence their actions with my own. So I could say something but I can't let them do stuff. They act as i suspect them to. But mostly I don't beliefe in myself to have such a big influence in other peoples action. Onetime a Lady

				change my actions.	attackt me and I could make her stop through my thoughts.

Table S5.6 Dream control 5

	What is the challenge in controlling... Write down the challenges you experience when trying to control the following item. Use a short explanation.			
Participant	… the objects in you dream?	… the dream environment in your dream?	… time in your dream?	… gravity in your dream?
1	Normalerweise gut, aber schwieriger desto komplexer der traumwelt ist	Einfach wenn es ein kopie ist und man es einwenig ändern will, aber Ultra schwierig ein ganzen Welt grundaufneu aufzubauen	Momentan unmöglich für mich…(Ausnahmen ein einzigmal, als ich geträumt habe, dass ein kind am erhängen ist und meine Beine feststeckten und ich nicht rechtzeitig zu hilfe eilen konnte.. musste ich jemand anders im traum kontrollieren und später die zeit für das kind einwenig zurück drehen.. damit er nicht erstickt)	Normalerweise einfach, aber schwieriger desto komplexer der traum ist
2	To believe that it is possible	To believe that it os possible	To believe that it is possible	Jump and being confident to not fall down again
3	making impossible functions possible	not really change something	relatively	its random
4	Mostly I can't imagine the objects to do stuff, only if it is possible or I can imagine it. Once I managed to steer a car on the track to where I wanted it to go.	I need to have a place or a image in my head for it to exist im my dream.	I never had influence in time because I never think about it in my dream.	As soon as I change something and do something supernatural like flying my dream gets unstable and I wake up.

Table S5.7 Dream control 6

	How much do the challenges you mentioned above affect the control over your dream? Rate the challenges for each item on a scale from 1-5. (1 - no effect on the dream control at all, 5 - affects the dream control extremely) the challenge with…

Participant	your own dream body	other dream characters' bodies	your own dream body movement	own dream actions	other dream characters' actions	the objects in you dream	the dream environment in your dream	time in your dream	gravity in your dream
1	1	1	1	1	1	2	2	5	4
2	5	3	5	5	3	5	4	3	5
3	1	1	2	5	3	4	5	1	2
4	2	1	4	4	1	2	2	1	4

Table S5.8 Dream control 7

	What are challenges you already managed to do in a lucid dream? (Select all that apply)						
Participant	Communicate with other people in your dream	Deliberately shape your environment	Flying with full control	Make day turn to night	Going through walls or things	Going through dream characters	Other
1	Yes	Yes	Yes	Yes	No	No	
2	Yes	No	Yes	Yes	Yes	No	Transform into animals
3	Yes	No	Yes	Yes	No	Yes	
4	Yes	Yes	Yes	No	No	No	
